# Photodynamic Diagnosis–guided Dual Laser Ablation for Upper Urinary Tract Carcinoma: Preoperative Preparation, Surgical Technique, and Clinical Outcomes

**DOI:** 10.1016/j.euros.2021.03.009

**Published:** 2021-04-23

**Authors:** Takashi Yoshida, Takashi Murota, Tomoaki Matsuzaki, Kazuyoshi Nakao, Chisato Ohe, Tadashi Matsuda, Hidefumi Kinoshita

**Affiliations:** a Department of Urology and Andrology, Kansai Medical University Hospital, Hirakata, Japan; b Department of Urology and Andrology, Kori Hospital, Kansai Medical University, Osaka, Japan; c Department of Urology and Andrology, General Medical Center, Kansai Medical University, Osaka, Japan; d Department of Pathology and Laboratory Medicine, Kansai Medical University, Osaka, Japan

**Keywords:** Diagnosis, Photodynamic diagnosis, Laser ablation, Ureteroscopy, Upper urinary tract carcinoma

## Abstract

**Background:**

Although ureteroscopic surgery (URS) is beneficial for low-risk upper urinary tract carcinoma (UTUC), there is no standardized URS technique or navigation system for challenging cases.

**Objective:**

To present a URS technique for UTUC using thulium (Tm):YAG and holmium (Ho):YAG lasers under photodynamic diagnosis (PDD) guidance, named PDD-guided dual laser ablation (PDD-DLA) and compare its efficacy with that of conventional Ho:YAG laser ablation (HLA; historical control).

**Design, setting, and participants:**

The study included ten consecutive UTUC patients who underwent PDD-DLA between 2017 and 2019. The control group comprised 16 consecutive patients who underwent HLA between 2006 and 2016.

**Surgical procedure:**

After oral administration of 5-aminolevulinic acid (20 mg/kg), UTUC tumors were endoscopically resected via PDD-DLA.

**Measurements:**

Clinical data were prospectively collected for our institutional UTUC data set. Disease progression, UTUC recurrence, and clinical outcomes were assessed.

**Results and limitations:**

PDD-DLA was successfully performed in all patients. The median tumor size was 23.5 mm (interquartile range [IQR] 12.8–30.0) and there were four cases (40.0%) of high-grade tumor. The median operative time was 120 min (IQR 98.5–142.5). No Clavien-Dindo grade ≥3 complications were observed. There were no differences in most clinical characteristics between the PDD-DLA and HLA groups. The 2-yr progression-free survival rate was 100% in the PDD-DLA group and 58.7% in the HLA group (*p* = 0.0197), and the 2-yr recurrence-free survival rate was 57.1% and 41.3%, respectively (*p* = 0.072). The PDD-DLA group had a lower incidence rate of salvage RNU compared with the HLA group (0.0% vs 50%; *p* = 0.009). The small sample size might affect the reproducibility of these results.

**Conclusions:**

PDD-DLA seems to be an effective and feasible endoscopic technique for UTUC treatment with favorable oncological outcomes.

**Patient summary:**

We investigated a new laser technique for treating cancer of the upper urinary tract called photodynamic diagnosis–guided dual laser ablation. Our strategy was effective in removing tumors and stopping bleeding. Further studies in larger groups of patients are needed to confirm whether this technique improves cancer outcomes.

## 1 Introduction

Although radical nephroureterectomy (RNU) is still the gold standard for treatment of upper tract urothelial carcinoma (UTUC), ureteroscopic surgery (URS) has become a more common treatment option for low-risk tumors (<2 cm, low grade, and low stage) [1]. Current guidelines also recommend URS for patients with solitary kidneys, bilateral tumors, or impaired renal function on a case-by-case basis [1]. However, in clinical practice, there are patients with noninvasive but larger/multifocal or high-grade UTUC who are ineligible for RNU for the following reasons: poor performance status, severe comorbidities, concomitant other types of progressive cancer, unable to receive general anesthesia, or refusal to undergo RNU. Therefore, there is an urgent need to expand URS indications using strategies that are safe and feasible for these patients.

Two possible approaches can be used: (1) precise and effective tumor ablation while controlling bleeding [2]; or (2) accurate identification of surgical margins and residual viable tumors, which may contribute to a reduction in the recurrence rate [3], [4]. Instead of a neodymium (Nd):yttrium aluminum garnet (YAG) laser, which is widely used for URS but not recommended for treating ureteral tumors owing to its deep penetration, a thulium (Tm):YAG laser, which has a strong hemostatic effect with shallow penetration, can be used. Furthermore, this can be combined with a conventional holmium (Ho):YAG laser, which has high resection efficacy [2], [3], [5]. In addition, photodynamic diagnosis (PDD) with oral 5-aminolevulinic acid (5-ALA) can be used to detect tumor margins, residual tumors, and even floating tumor cells [6], [7]. We hypothesized that the combined use of these devices could greatly improve oncological outcomes for UTUC patients undergoing URS.

The aim of this study was to describe our technique, PDD-guided dual laser ablation (PDD-DLA), in patients with noninvasive UTUC. To evaluate its clinical efficacy, we compared two URS techniques, PDD-DLA versus conventional Ho:YAG laser ablation (HLA, historical control), in terms of surgical and oncological outcomes.

## 2 Patients and methods

### 2.1 Patients

The study included ten consecutive patients with noninvasive UTUC who underwent PDD-DLA between July 2017 and September 2019. The data were collected prospectively and reviewed retrospectively. As a historical control group, 16 consecutive patients with UTUC treated with HLA between September 2006 and October 2016 were included. This retrospective study was approved by the ethics board of Kansai Medical University (IRB no. 2018036). The inclusion criteria were determined according to the 2015 European Association of Urology and 2014 Japanese Urological Association guidelines as follows: elective cases: unifocal, low grade, tumor size ≤1 cm, and no evidence of infiltrative tumor on imaging; and imperative cases: solitary kidney, bilateral tumors, or insufficient kidney function [8], [9]. In addition, we performed URS for patients with noninvasive UTUC, including those who were older (≥80 yr), those with poor performance status, those with severe comorbidities, those with other aggressive tumors, and those who refused RNU regardless of tumor grade, size, and multifocality (ie, relative cases) according to a previous study [10].

### 2.2 Preoperative preparation and investigational agent

At the initial visit, patients underwent a general work-up, including computerized tomography (CT) ± urography (CT-U, if possible), cystoscopy, and urine cytology. Diagnostic URS with biopsy was then performed to evaluate the tumor stage, architecture, and histology. Pre-stenting was an option for future URS in patients with ureter narrowing. On the basis of the clinical and pathological findings, a final decision on URS was made. On the day of surgery, each patient received oral 5-ALA at 20 mg/kg (SBI Pharmaceuticals, Tokyo, Japan) dissolved in 50 ml of water 1 h before URS.

### 2.3 General setting and surgical apparatus for URS

**Fig. 1:**
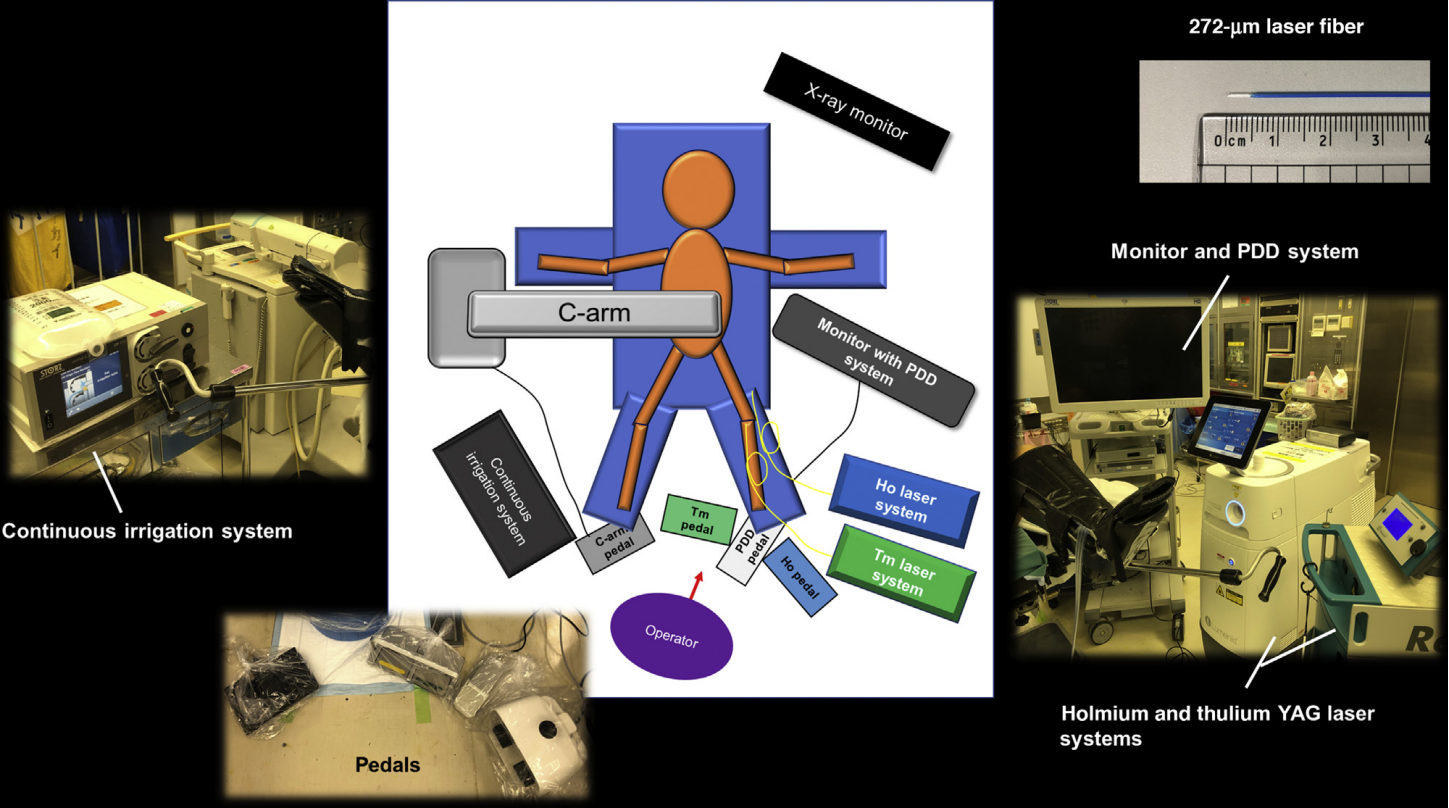
Patient positioning and surgical devices. PDD = photodynamic diagnosis.

URS was performed under general or lumbar anesthesia in the lithotomy position. All PDD-DLA procedures were performed by a single endourologist (T.Y). The general setup is shown in Figure 1. A D-Light C system (Karl Storz, Tuttlingen, Germany) and a protoporphyrin IX excitation eyepiece filter permitting blue-violet light (SBI Pharmaceuticals, Tokyo, Japan) were used for PDD. URF-P6 or P7 (Olympus, Tokyo, Japan) and Ultrathin 6-Fr (Richard Wolf, Knittlingen, Germany) ureteroscopes were used. A UROMAT E.A.S.I. SCB device (Karl Storz) was used to adjust irrigation pressure levels to obtain an adequate irrigation flow according to the surgical procedure. Tumor extraction was performed with an N-Circle nitinol tipless stone extractor (Cook Medical, Bloomington, IN, USA). Piranha forceps (Boston Scientific, Marlborough, MA, USA) were used to perform ureteroscopic biopsy. A Revolix120 Tm:YAG laser system (LISA Laser Products, Katlenburg-Lindau, Germany) and Lumenis Pulse 120H Ho:YAG laser system (Lumenis, Yokneam Illit, Israel) were used for ablation. The laser settings were as follows: 5 W (left pedal) and 15 W (right pedal) for Tm:YAG; and 0.4 J/15 Hz in the long-pulse mode (left pedal) and 1 J/10 Hz in the short-pulse mode (right pedal) for Ho:YAG. For both laser systems, 272-μm laser fibers were used; the outer cover was peeled off to expose the tip of the quartz part (5 mm) to indicate the penetration depth (Fig. 1). For patients with proximal ureteral or renal pelvic tumors, a ureteral access sheath (10/12-, 11/13-, or 12/14-Fr) was routinely placed.

### 2.4 URS with PDD-DLA

Each procedure began with placement of a guide wire up to the ureteropelvic junction under direct vision with a semi-rigid ureteroscope or flexible ureteroscope while avoiding bleeding from the tumors and urinary wall. A semi-rigid ureteroscope was used for distal or middle ureteral tumors, and a flexible ureteroscope with a ureteral access sheath was used for proximal ureteral or renal pelvic tumors.

Subsequent procedures were performed according to tumor size (≤5 mm or >5 mm).

#### 2.4.1 Tumors ≤5 mm

For tumors ≤5 mm, ablation with the Tm: YAG laser was first performed to coagulate the entire tumor through contact with its surface. When the tumor was adequately coagulated, Ho:YAG laser ablation was conducted to resect the tumor tissues. Then PDD was performed to detect residual tumors and, if present, additional laser ablation was carried out until all PDD-positive tumors were eradicated.

#### 2.4.2 Tumors >5 mm

**Fig. 2:**
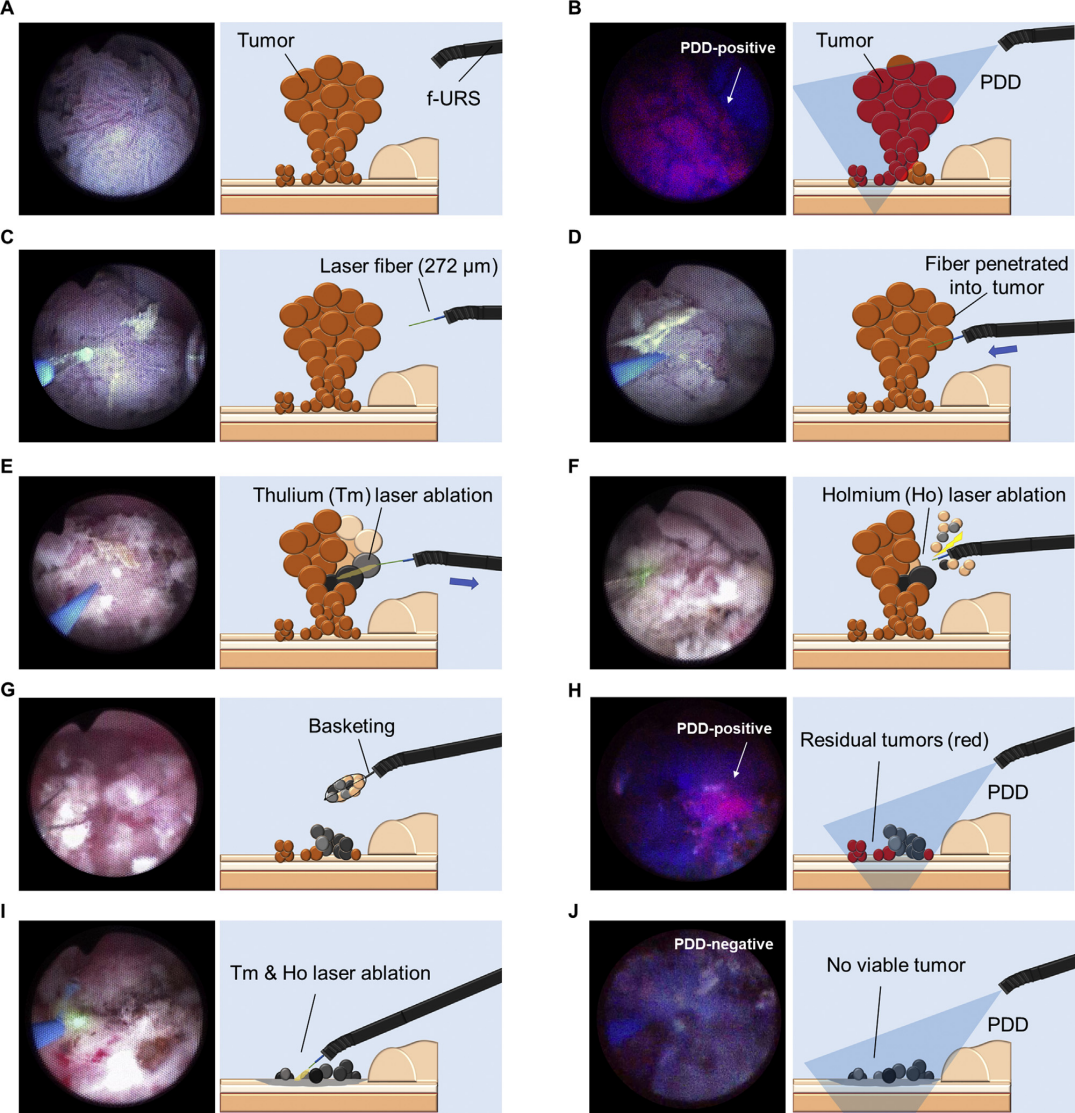
Surgical flow for photodynamic diagnosis (PDD)-guided dual laser ablation of renal pelvic tumors. (A) Examine the tumor under white light. (B) Examine the tumor spread with PDD. (C) Set the laser fiber as appropriate. (D) Penetrate the tumor with the laser fiber. (E) Ablate the tumor with a 15-W thulium:YAG laser while pulling it out, and repeat this procedure until the tumor becomes ischemic. (F) Resect the ischemic tumor using a holmium:YAG laser. (G) Remove the tumor fragments using a stone basket. (H) Identify the surgical margins and residual microtumors with PDD. (I) Ablate and resect residual tumors with both lasers. (J) Confirm that there are no PDD-positive tumors. f-URS = flexible ureteroscope.

**Fig. 3:**
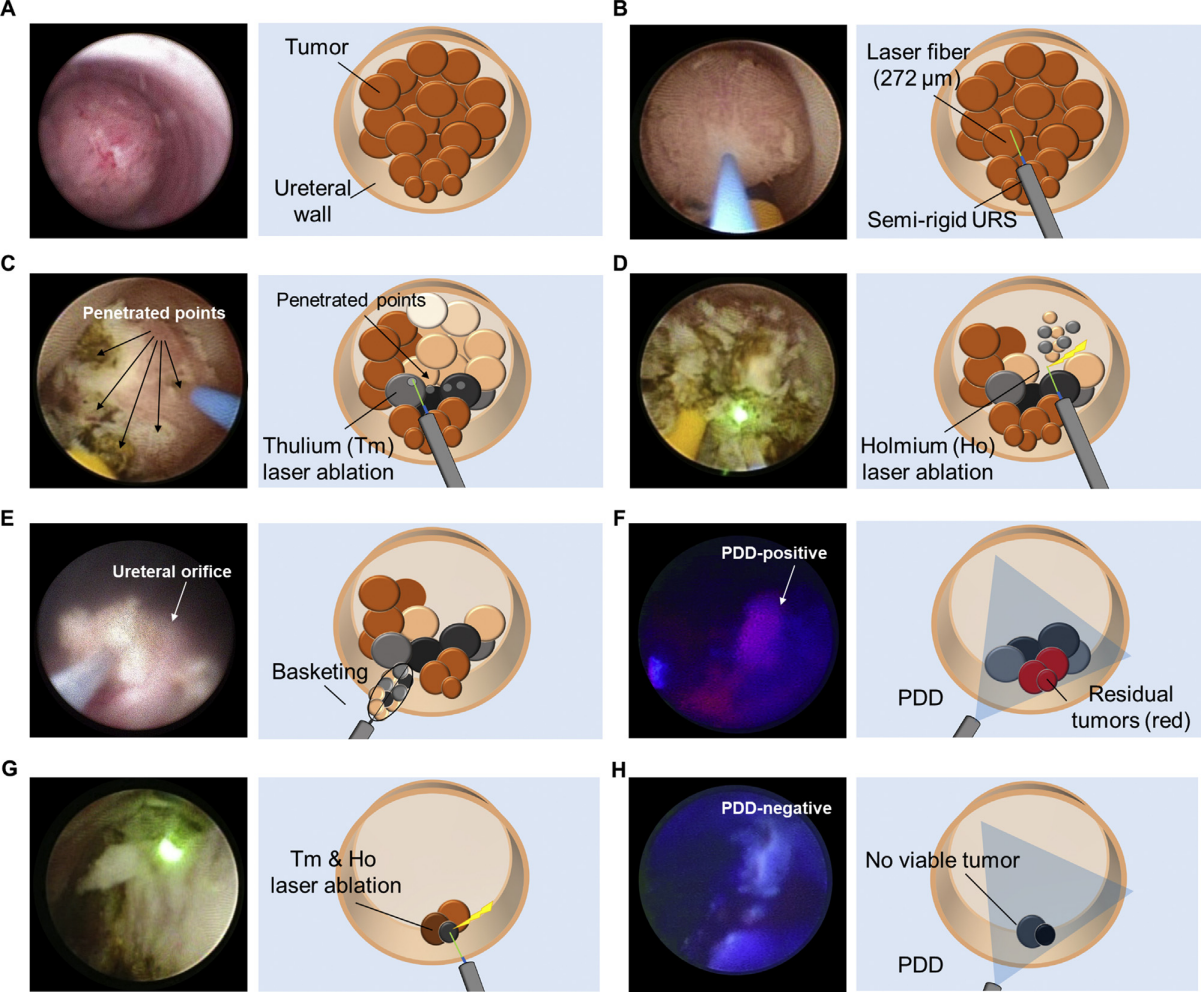
Surgical flow for photodynamic diagnosis (PDD)-guided dual laser ablation of ureteral tumors. (A) Examine the tumor under white light. (B) Penetrate the tumor with a laser fiber. (C) Ablate the tumor with a thulium laser. (D) Resect the ischemic tumor using a holmium:YAG laser. (E) Remove the tumor fragments using a stone basket. (F) Identify the surgical margins and residual microtumors with PDD. (G) Ablate and resect residual tumors with both lasers. (H) Confirm that there are no PDD-positive tumors. URS = ureteroscope.

For tumors >5 mm (renal pelvis Fig. 2A–J; ureter Fig. 3A–H), the laser was used to penetrate the tumor with the length of the quartz part as guidance (5 mm), and intratumor ablation with a 15-W Tm:YAG laser was performed while slowly pulling out the laser fiber. When the target tumor was close to the urinary tract wall, the laser energy was reduced to 5 W. After repeating this procedure until the tumor became ischemic, Ho:YAG laser ablation was carried out to resect the coagulated tumor tissues. The tumor fragments were removed using an extraction basket. The same procedures were repeated until the tumors were significantly reduced. Then PDD was performed to detect residual tumors and surgical margins. Lesions that were suspicious on PDD were ablated with the Tm:YAG and Ho:YAG lasers to ensure that all PDD-positive lesions were completely ablated.

When the operative time was ≥3 h, a staged URS procedure was considered. At the end of the procedure, a 6-Fr ureteral stent and a 14-Fr urethral catheter were inserted.

### 2.5 Postoperative course and follow-up schedule

All patients were instructed to avoid exposure to direct sunlight, brightly focused indoor light, or strong light sources for 48 h. The urethral catheter was removed on postoperative day 1.

At 4 wk after URS, CT-U was performed to detect any disease progression or significant recurrence. If deterioration was observed on imaging, immediate RNU was strongly recommended. If not, a second-look URS was scheduled for 6 wk after the URS procedure. Second-look URS was performed using a flexible ureteroscope without PDD (after laser ablation, inflamed mucosa often cause PDD false positives) and biopsy samples were obtained from the ablated area. When there was no evidence of malignancy, follow-up was performed according to the regular protocol: CT (or CT-U), cytology, and cystoscopy every 3 mo, with ureteroscopy every 6 mo for 2 yr or more.

### 2.6 Data collection and statistical analysis

The endpoints of this study were progression-free survival (PFS; disease progression was defined as the occurrence of distant metastases on imaging, relapsing tumors that could not be controlled with a conservative approach, or upgrading from low to high grade in elective cases) and recurrence-free survival (RFS; UTUC recurrence was defined as a relapsing tumor determined ureteroscopically or radiologically in the same renal unit as the primary tumor). In addition, intravesical recurrence after URS was assessed. Tumor stage and grade were evaluated according to the 2010 TNM staging system and the 2016 World Health Organization consensus classification, respectively [11], [12]. Complications related to URS were evaluated according to the Clavien-Dindo system [13]. Clinicopathological variables were compared between the two surgical types using χ^2^ and Mann-Whitney *U* tests. Survival analysis was performed using the Kaplan-Meier method with the log-rank test. Analysis of covariance with adjustment for the baseline preoperative estimated glomerular filtration rate (eGFR) was used for comparing the percentage change in eGFR at 12 mo or the last visit between the groups. All statistical analyses were performed using EZR version 1.37 (Saitama Medical Center, Jichi, Japan) [14]. A two-sided *p* value of < 0.05 was considered statistically significant.

## 3 Results

The clinicopathological data are summarized in Table 1. Overall, the variables were statistically well balanced between the two groups, except for baseline eGFR. In the PDD-DLA group, the median tumor size was 23.5 mm (interquartile range 12.8–30.0), and the number of cases with high-grade tumors was eight (80.0%). In addition, the number of imperative/relative cases was eight (80.0%). Two patients (20.0%) in the PDD-DLA group subsequently died of non-UTUC disease within the 1-yr follow-up period.

**Table 1:** Clinicopathological characteristics

Variable	PDD-guided dual LA	Ho:YAG LA (historical control)	*p* value
	(*n* = 10)	(*n* = 16)	
Median age, yr (IQR)	77.0 (71.3–83.3)	78.5 (72.8–80.3)	0.792
Sex, *n* (%)			0.683
Female	3 (30.0)	7 (43.8)	
Male	7 (70.0)	9 (56.2)	
ECOG PS, *n* (%)			0.124
0	6 (60.0)	3 (18.8)	
1	1 (10.0)	4 (25.0)	
2	3 (30.0)	5 (31.2)	
3	0 (0.0)	4 (25.0)	
ASA score, *n* (%)			0.876
1	4 (40.0)	4 (25.0)	
2	3 (30.0)	7 (43.8)	
3	3 (30.0)	5 (31.2)	
Median baseline eGFR, ml/min (IQR)	67.5 (59.3–69.5)	46.0 (29.5–55.8)	0.031
Median tumor size, mm (IQR)	23.5 (12.8–30.0)	16.50 (10.0–28.5)	0.351
Tumor site, *n* (%)			0.234
Renal pelvis	5 (50.0)	4 (25.0)	
Ureter	5 (50.0)	12 (75.0)	
Multifocality, *n* (%)			0.352
Single	9 (90.0)	11 (68.8)	
Multiple	1 (10.0)	5 (31.2)	
History of bladder cancer, *n* (%)			1.000
No	6 (60.0)	9 (56.2)	
Yes	4 (40.0)	7 (43.8)	
Clinical stage, *n* (%)			1.000
TaN0M0	9 (90.0)	14 (87.5)	
T1N0M0	1 (10.0)	2 (12.5)	
Tumor grade on URS biopsy, *n* (%)			0.391
Low	6 (60.0)	5 (31.2)	
High	4 (40.0)	9 (56.2)	
Unverified	0 (0.0)	2 (12.5)	
Indications for URS, *n* (%)			0.419
Selective case	2 (20.0)	2 (12.5)	
Imperative/relative case	8 (80.0)	14 (87.5)	

ASA = American Society of Anesthesiologists; eGFR = estimated glomerular filtration rate; ECOG PS = Eastern Cooperative Oncology Group performance status; URS = ureteroscopic surgery; IQR = interquartile range; LA = laser ablation; PDD = photodynamic diagnosis.

The median operative time was relatively longer in the PDD-DLA group than in the HLA group (120.0 vs 74.5 min; *p* = 0.097). PDD-DLA was successfully performed in all ten patients (100.0%), whereas the conventional technique was successful in 13 patients (81.3%). Staged procedures were required for three patients (30.0%) patients who had renal pelvic tumor(s) ≥3 cm in the PDD-DLA group (two sessions for two patients and four sessions for one patient); however, no severe complications (Clavien-Dindo grade ≥3) were observed. One patient (6.3%) in the HLA group developed postoperative ureteral stricture requiring endoscopic balloon dilatation (Table 2). The rate of salvage RNU was lower for the PDD-DLA group than for the HLA group (0.0% vs 50%; *p* = 0.009). All patients who required salvage RNU had imperative/relative indications. After adjustment for the baseline value, the percentage change in eGFR from baseline showed a greater decreasing trend in the HLA group than in the PDD-DLA group (*p* = 0.075; Table 2).

**Table 2:** Surgical and functional outcomes

Variable	PDD-guided dual LA	Ho:YAG LA (historical control)	*p* value
	(*n* = 10)	(*n* = 16)	
Median OT for initial procedure, min (IQR)	120.0 (98.5–142.5)	74.5 (50.8–135.3)	0.097
Primary lesion cleared with URS, *n* (%)	10 (100.0)	13 (81.3)	0.262
Cleared with one procedure	7 (70.0)	10 (62.5)	0.508
Cleared with staged procedures	3 (30.0)	3 (18.8)	
Clavien-Dindo complications, *n* (%)			0.77
Grade 1	9 (90.0)	12 (75.0)	
Grade 2	1 (10.0)	3 (18.8)	
Febrile urinary tract infection	1 (10.0)	3 (18.8)	
Grade 3a	0 (0.0)	1 (6.2)	
Ureteral stricture	0 (0.0)	1 (6.2)	
Grade 3b/4a/4b/5	0 (0.0)	0 (0.0)	
Salvage radical nephroureterectomy, *n* (%)			0.009
Not required	10 (100.0)	8 (50.0)	
Required	0 (0.0)	8 (50.0)	
Median postoperative eGFR, ml/min (IQR) a	70.5 (47.3–80.5)	33.0 (23.3–48.0)	0.014
Median change in eGFR from baseline, % (IQR)	5.7 (2.9–18.9)	−11.4 (−23.1 to −1.0)	0.075 b

eGFR = estimated glomerular filtration rate; IQR = interquartile range; LA = laser ablation; OT = operation time; PDD = photodynamic diagnosis; URS = ureteroscopic surgery.

a At 1 yr or last visit after URS.

b Adjusted for baseline eGFR.

**Fig. 4:**
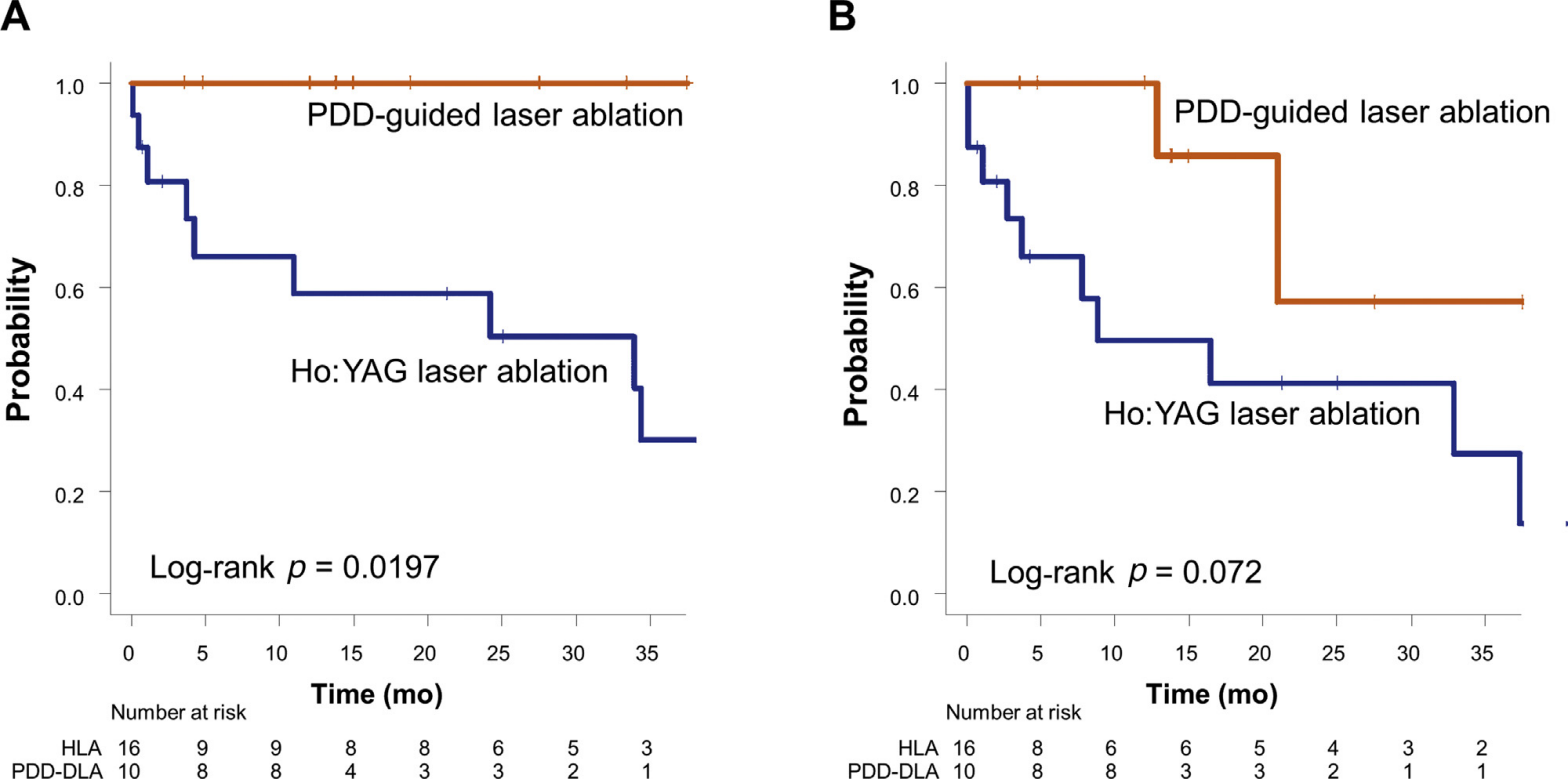
Oncological outcomes for upper tract urothelial carcinoma. (A) Progression-free survival and (B) recurrence-free survival. HLA = holmium laser ablation; PDD-DLA = photodynamic diagnosis–guided dual laser ablation.

Regarding oncological outcomes, the 2-yr PFS rate was significantly higher among patients treated with PDD-DLA than in the HLA group (100% vs 58.7%; *p* = 0.0197; Fig. 4A). The 2-yr RFS rate tended to be better in the PDD-DLA group compared to those treated with HLA (57.1% vs 41.3%; *p* = 0.072; Fig. 4B). In addition, the 2-yr intravesical RFS rate was not significantly different between the two groups, although it was higher in the PDD-DLA group (85.7% vs 44.6%; *p* = 0.087).

## 4 Discussion

In the present study, we demonstrated that PDD-DLA provided better oncological outcomes compared with conventional HLA in terms of disease progression and UTUC recurrence. Moreover, the PDD-DLA group had a lower incidence rate of salvage RNU compared to the HLA group. Therefore, PDD-DLA may be a feasible surgical method for resection of noninvasive UTUCs, even in challenging cases.

A Ho:YAG laser alone or combined with Ho:YAG and Nd: YAG lasers is traditionally used for URS in UTUC [2], [4], [15]. The Tm:YAG laser system, which provides maximum hemostasis and coagulation with shallow penetration (0.4 mm), has been increasingly used for endoscopic treatment of UTUC since Defidio et al first reported its use in 2011 [3], [5], [16], [17], [18]. We agree with Defidio et al that combined use of Ho:YAG and Tm:YAG lasers (not a Tm:YAG laser alone) may lead to less bleeding and shorter operating times, thus increasing the possibility of treating larger tumors [5], [19]. However, in our experience, Tm:YAG laser use for coagulation is difficult for the deeper parts of tumors owing to its shallow penetration depth, necessitating frequent switching between the Tm:YAG and Ho:YAG lasers for coagulation and resection, respectively. To overcome this problem, we inserted the laser fiber into the tumor and performed intratumor Tm:YAG laser ablation, which can rapidly reduce intratumor blood flow and make the tumor “stone-like”. This procedure facilitates faster tumor resection when performing subsequent Ho:YAG laser ablation because of the lowered risk of bleeding [20].

Another advantage of the intratumor laser ablation technique is that it can avoid direct temperature increases, cavitation induced by Tm:YAG laser ablation of the urinary tract wall, and missed ablation due to respiratory fluctuations, especially in obstructive ureteral or renal pelvic tumors. Therefore, we can initiate resection at the distal portion of the tumor and proceed to work proximally, even though we cannot perform URS beyond the tumor. Indeed, we did not observe any ureteral stricture and completed all procedures regardless of tumor size (Table 2). However, to avoid unexpected urinary tract perforation or mucosal injury due to this blind technique, intratumor ablation must be performed by delicately adjusting the penetration depth of the 5-mm exposed quartz part of the laser fiber while understanding the actual tumor size and its anatomical morphology.

According to previous studies in UTUC, the 2-yr estimated PFS rate was approximately 70%, whereas the 2-yr estimated RFS rate ranged from 10% to 40% in cohorts including imperative/relative cases [4], [5], [15]. Such high recurrence rates are consistent with the findings of Villa et al [3], who detected UTUCs in 51.2% of cases on second-look URS, and 85.7% of these were at the same lesion as the primary tumor. These findings suggest that surgeons may have difficulty in detecting residual tumors that are incompletely ablated or microlesions under white light. Therefore, we introduced a navigation system to accurately identify residual tumor and surgical margins.

There are several diagnostic tools for UTUC, and narrow-band imaging (NBI) and PDD may be possible during URS [21]. However, when considering the mechanism of NBI in terms of the specific light absorption of hemoglobin and the mucosa, this method cannot be used for ablated or ischemic tumor tissues. Nevertheless, for PDD, fluorescence emission using blue light remains feasible even in isolated exfoliated cells if protoporphyrin IX has accumulated in the tumor cells [7]. A recent study demonstrated that compared with white-light URS, PDD-URS had significantly higher sensitivity (93.8% vs 62.5%; *p* = 0.0025) and accuracy (0.86 vs 0.75; *p* = 0.0297) in the detection of UTUCs, including non-apparent lesions [6].

Despite the small sample size and short follow-up period, there were only two cases (20.0%) of recurrence in which the tumors were different from the primary lesion in the PDD-DLA group. Therefore, PDD might greatly contribute to complete resection of the primary tumor by confirming the depth and spread of UTUC regardless of tumor size and grade, leading to favorable oncological outcomes. Attenuation of the fluorescence emission during the URS procedure can be reduced by controlling the timing of 5-ALA administration, and PDD-related tangential artifacts can be minimized by using a closely directed f-URS and deepening the approach angle to the pelvis or ureteral wall [6].

This study has some limitations. First, the small sample size and short follow-up period preclude clinically significant conclusions. Second, patients in the historical control group were treated by multiple surgeons with different levels of experience and various surgical devices in each time frame, possibly resulting in bias. Third, oral 5-ALA for UTUC is only available in some countries. Fourth, we could not use digital URS, which provides high-quality visibility, because digital URS with a protoporphyrin IX excitation eyepiece filter for PDD is unavailable. Fifth, we could not compare the cost-effectiveness of PDD-DLA with that of the conventional method because our procedure was performed as part of a clinical trial in which drugs were provided free of charge. Finally, we did not routinely use pre- or postoperative instillation as adjuvant therapy. In the future, perioperative instillation of a gel formulation of mitomycin C with URS could be considered as a novel kidney-sparing strategy [22].

## 5 Conclusions

We presented a step-by-step description of our PDD-DLA technique for noninvasive treatment of UTUC, including challenging cases. Use of an appropriate combination of lasers, techniques, and surgical navigation systems may contribute to better oncological outcomes and expand the indications for endoscopic management.

  ***Author contributions:*** Takashi Yoshida had full access to all the data in the study and takes responsibility for the integrity of the data and the accuracy of the data analysis.

  *Study concept and design:* Yoshida.

*Acquisition of data:* Yoshida, Murota, Matsuzaki, Nakao.

*Analysis and interpretation of data:* All authors.

*Drafting of the manuscript:* Yoshida.

*Critical revision of the manuscript for important intellectual content:* All authors.

*Statistical analysis:* Yoshida.

*Obtaining funding:* Yoshida.

*Administrative, technical, or material support:* None.

*Supervision:* Yoshida, Murota, Matsuda, Kinoshita.

*Other:* None.

  ***Financial disclosures:*** Takashi Yoshida certifies that all conflicts of interest, including specific financial interests and relationships and affiliations relevant to the subject matter or materials discussed in the manuscript (eg, employment/affiliation, grants or funding, consultancies, honoraria, stock ownership or options, expert testimony, royalties, or patents filed, received, or pending), are the following: None.

  ***Funding/Support and role of the sponsor:*** This study was supported by the KAKENHI fund of the 10.13039/501100001691Japan Society for the Promotion of Science (grant no. 20K07601 to T.Y.). The sponsor played a role in preparation of the manuscript.

  ***Acknowledgments:*** The authors express their heartfelt gratitude to SBI Pharmaceuticals for supplying the investigational drug in a previous prospective study. They also thank SBI Pharmaceuticals, MC Medical, Karl Storz Endoscopy Japan, and Takai Medical for their technical assistance with the surgical devices.

## References

[1] Rouprêt M., Babjuk M., Burger M. (2021). European Association of Urology guidelines on upper urinary tract urothelial carcinoma: 2020 update. Eur Urol.

[2] Bagley D.H., Grasso M. (2010). Ureteroscopic laser treatment of upper urinary tract neoplasms. World J Urol.

[3] Villa L., Cloutier J., Letendre J. (2016). Early repeated ureteroscopy within 6–8 weeks after a primary endoscopic treatment in patients with upper tract urothelial cell carcinoma: preliminary findings. World J Urol.

[4] Shvero A., Abu-Ghanem Y., Laufer M. (2021). Endoscopic treatment for large multifocal upper tract urothelial carcinoma. J Urol.

[5] Yoshida T., Taguchi M., Inoue T., Kinoshita H., Matsuda T. (2018). Thulium laser ablation facilitates retrograde intra-renal surgery for upper urinary tract urothelial carcinoma. Int J Urol.

[6] Yoshida T., Setsuda S., Ishizuka M., Inoue T., Kinoshita H., Matsuda T. (2020). Photodynamic diagnosis with oral 5-aminolevulinic acid for upper urinary tract carcinoma: a prospective clinical trial. J Endourol.

[7] Miyake M., Nakai Y., Anai S. (2014). Diagnostic approach for cancer cells in urine sediments by 5-aminolevulinic acid-based photodynamic detection in bladder cancer. Cancer Sci.

[8] Rouprêt M., Babjuk M., Compérat E. (2015). European Association of Urology guidelines on upper urinary tract urothelial cell carcinoma: 2015 update. Eur Urol.

[9] Oya M., Kikuchi E., Committee for Establishment of Clinical Practice Guideline for Management of Upper Tract Urothelial Carcinoma, Japanese Urological Association (2015). Evidenced-based clinical practice guideline for upper tract urothelial carcinoma (summary – Japanese Urological Association, 2014 edition). Int J Urol.

[10] Cutress M.L., Stewart G.D., Wells-Cole S., Phipps S., Thomas B.G., Tolley D.A. (2012). Long-term endoscopic management of upper tract urothelial carcinoma: 20-year single-centre experience. BJU Int.

[11] Edge SB, Compton CC (2010). The American Joint Committee on Cancer: the 7th edition of the AJCC cancer staging manual and the future of TNM. Ann Surg Oncol.

[12] Moch H., Humphrey P.A., Ulbright T.M., Reuter V.E. (2016). WHO classification tumors of the urinary system and male genital organs.

[13] Dindo D., Demartines N., Clavien P.A. (2004). Classification of surgical complications: a new proposal with evaluation in a cohort of 6336 patients and results of a survey. Ann Surg.

[14] Kanda Y. (2013). Investigation of the freely available easy-to-use software ‘EZR’ for medical statistics. Bone Marrow Transplant.

[15] Villa L., Haddad M., Capitanio U. (2018). Which patients with upper tract urothelial carcinoma can be safely treated with flexible ureteroscopy with holmium:YAG laser photoablation? Long-term results from a high volume institution. J Urol.

[16] Musi G., Mistretta F.A., Marenghi C. (2018). Thulium laser treatment of upper urinary tract carcinoma: a multi-institutional analysis of surgical and oncological outcomes. J Endourol.

[17] Defidio L., De Dominicis M., Di Gianfrancesco L., Fuchs G., Patel A. (2011). First collaborative experience with thulium laser ablation of localized upper urinary tract urothelial tumors using retrograde intra-renal surgery. Arch Ital Urol Androl.

[18] Bozzini G., Gastaldi C., Besana U. (2021). Thulium-laser retrograde intra renal ablation of upper urinary tract transitional cell carcinoma: an ESUT Study. Minerva Urol Nephrol.

[19] Defidio L., Antonucci M., De Dominicis M., Fuchs G., Patel A. (2019). Thulium-holmium:YAG duo laser in conservative upper tract urothelial cancer treatment: 13 years experience from a tertiary national referral center. J Endourol.

[20] Proietti S, Rodríguez-Socarrás ME, Eisner BH, et al. Thulium:YAG versus holmium:YAG laser effect on upper urinary tract soft tissue: evidence from an ex vivo experimental study. J Endourol. In press. 10.1089/end.2020.0222. 32808543

[21] Knoedler JJ, Raman JD (2018). Advances in the management of upper tract urothelial carcinoma: improved endoscopic management through better diagnostics. Ther Adv Urol.

[22] Kleinmann N., Matin S.F., Pierorazio P.M. (2020). Primary chemoablation of low-grade upper tract urothelial carcinoma using UGN-101, a mitomycin-containing reverse thermal gel (OLYMPUS): an open-label, single-arm, phase 3 trial. Lancet Oncol.

